# Morphea on the breast and pregnancy

**DOI:** 10.11604/pamj.2013.16.22.3317

**Published:** 2013-09-18

**Authors:** Loubna Benchat, Fatima Zahra Mernissi

**Affiliations:** 1 CHU Hassan II, Departement of Dermatology, Fes, Morocco

**Keywords:** Morphea, dermal fibrosis, pregnancy

## Image in medicine

Morphea is an uncommon, indolent, dermatologic disorder characterized by dermal fibrosis and collagen deposition. Its appearance during pregnancy is rare. We report a case of 34-year-old woman, 3 months pregnant, presented with a two-month history of localized skin changes on the left breast. The lesion gradually increased in size. On examination, we noticed an atrophic and retracted left breast with skin changes represented by pigmentation and sclerosis sparing the areola. A skin biopsy confirmed the diagnosis. The patient was treated with topical steroids with good improvement. When morphea affect the breast, it can easily be mistaken for malignant inflammatory breast disorders, like our patient. Previous case reports of breast-associated morphea have been described in the literature. They suggest a link with silicone breast implants, trauma, and external beam radiation for the treatment of breast cancer. None of these factors was found in our patient. To our knowledge, the pregnancy has never been reported as predisposing factor of the localization of morphea on the breast. The underlying etiology of morphea is unknown. Trauma/radiation, medications, infection, autoimmunity, and microchimerism have been described as associated factors with morphea. Thus pregnancy might be a predisposing factor of morphea because of the microchimerism. Indeed, chimeric cells are none self-cells transferred from fetus to mother during pregnancy. In addition, the pregnancy can alter the disease course of autoimmune diseases, including localized scleroderma.

**Figure 1:**
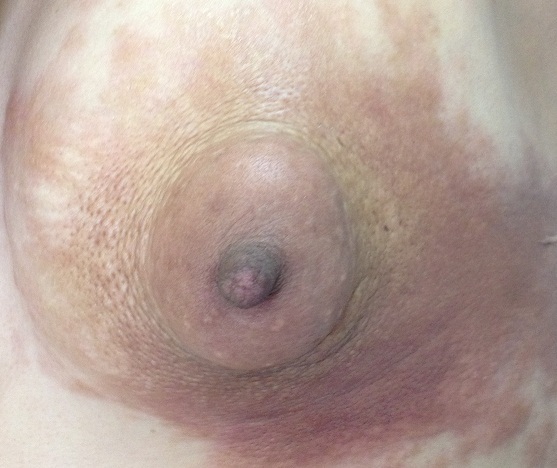
Retracted left breast with skin changes represented by atrophy, pigmentation and sclerosis

